# Low oxygen post conditioning prevents thalamic secondary neuronal loss caused by excitotoxicity after cortical stroke

**DOI:** 10.1038/s41598-019-39493-8

**Published:** 2019-03-19

**Authors:** Giovanni Pietrogrande, Katarzyna Zalewska, Zidan Zhao, Mahmoud Abdolhoseini, Wei Zhen Chow, Sonia Sanchez-Bezanilla, Lin Kooi Ong, Sarah J. Johnson, Michael Nilsson, Frederick R. Walker

**Affiliations:** 1 School of Biomedical Sciences and Pharmacy, University of Newcastle, Newcastle, Australia; 2 Hunter Medical Research Institute, Newcastle, Australia; 3 School of Electrical Engineering and Computing, University of Newcastle, Newcastle, Australia; 4 NHMRC Centre of Research Excellence Stroke Rehabilitation and Brain Recovery, Newcastle, Australia; 5 Priority Research Centre for Stroke and Brain Injury, University of Newcastle, Newcastle, Australia

## Abstract

In the current study, we were interested in investigating whether Low oxygen post-conditioning (LOPC) was capable of limiting the severity of stroke-induced secondary neurodegeneration (SND). To investigate the effect of LOPC we exposed adult male C57/BL6 mice to photothrombotic occlusion (PTO) of the motor and somatosensory cortex. This is known to induce progressive neurodegeneration in the thalamus within two weeks of infarction. Two days after PTO induction mice were randomly assigned to one of four groups: (i) LOPC-15 day exposure group; (ii) a LOPC 15 day exposure followed by a 15 day exposure to normal atmosphere; (iii) normal atmosphere for 15 days and (iv) normal atmosphere for 30 days (n = 20/group). We observed that LOPC reduced the extent of neuronal loss, as indicated by assessment of both area of loss and NeuN^+^ cell counts, within the thalamus. Additionally, we identified that LOPC reduced microglial activity and decreased activity within the excitotoxic signalling pathway of the NMDAR axis. Together, these findings suggest that LOPC limits neuronal death caused by excitotoxicity in sites of secondary damage and promotes neuronal survival. In conclusion, this work supports the potential of utilising LOPC to intervene in the sub-acute phase post-stroke to restrict the severity of SND.

## Introduction

Ischemic stroke is caused by the sudden interruption of the blood supply to the CNS. In most instances this interruption leads to neuronal loss and impaired brain function^1^. While the severity of the damage induced by stroke can be greatly reduced through hyper-acute interventions such as thrombolysis and thrombectomy, it is nearly always the case that patients are left with some degree of functional impairment. Currently, there are very few effective treatments available that are capable of reversing these persistent deficits. Adding to this challenge, research has identified secondary neurodegeneration (SND) as a related and significant problem with high clinical relevance^2^. SND is described as the progressive loss of neurons at sites that were connected to the infarct site but not directly impacted at the time of infarction.

While the processes driving SND are not yet well understood, it is clear that one of the mechanisms involves the spread of death through white matter tracts^3^. Currently, several experimental studies have demonstrated that a likely contributor to the spread of damage is glutamate mediated excitotoxicity^4,5^. While the process is relatively complex, its core is identified by the presence of excess glutamate that cannot be efficiently processed. The consequence of improper glutamate clearance can lead to the excessive stimulation of the ionotropic N-methyl-D-aspartate (NMDARs) receptors, massive influx of Ca^2+^ and unregulated intracellular signalling, which is known to ultimately cause cell death. One specific pathway of note in glutamate mediated excitotoxcity is the interaction of NMDARs with the scaffold postsynaptic density protein 95 (PSD-95). PSD-95 facilitates the coupling of NMDAR’s with neuronal nitric oxide synthetise (nNOS), which in turn can result in the production of nitric oxide (NO), which contributes to the formation of peroxynitrite, a compound that can drive cell death by apoptosis or necrosis^6–8^.

While inhibiting NMDAR activity has been of interest because of its potential to limit excitotoxicity, modulation has been difficult because of the central role that this receptor plays in normal CNS function^9^. To overcome this challenge, it has been demonstrated that suppressing PSD-95 binding to NMDAR acutely post-stroke can restrict excitotoxicity without markedly influencing NMDA activity^10^, such effects, however have not been demonstrated over longer time frames.

Recently, our team has been exploring the therapeutic potential of low oxygen post conditioning (LOPC). As a therapy LOPC has several advantages including that it has a well-characterised and acceptable safety profile^11^. Moreover, LOPC has already been shown in experimental stroke models to reduce infarct size^12^, promote neurogenesis and limit stroke induced deficits in motor^13,14^ and memory impairment^15,16^. More pertinent to the context of SND, a recent study showed that exposure to 8% oxygen for 5 days post-stroke reduces thalamic atrophy in a model of MCAO^17^.

**Figure 1:**
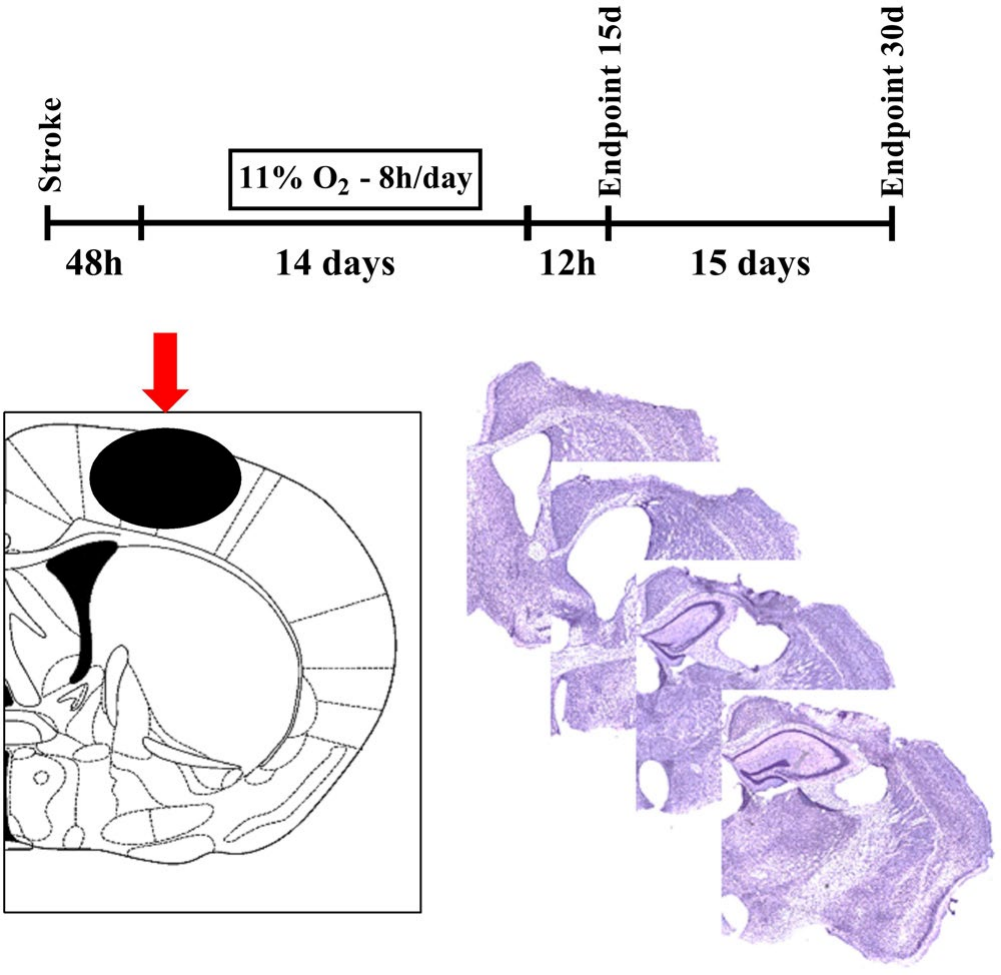
Experimental plan. Layout of the experimental design of LOPC and follow up protocol. Diagram illustrating the site of phototrombotic stroke induction (red arrow) at bregma 0 mm adapted from^47^ and representative cresyl violet stained coronal sections of a PTO stroke-affected hemisphere showing somatosensory cortex ablation (Bregma −0.6 to +1.5).

In the current study, we aimed to examine if the neuroprotective actions LOPC could be underpinned by the interventions ability to modulate NMDA-PSD-95-nNOS signalling. To consider if LOPC modulated NMDAR mediated excitotoxicity we chose an experimental model of photothrombotic (PT) ischemia targeting the motor-cortex. We considered this model the most suitable as it induces a highly focal infarct region and as such degenerative effects occurring at distance are only driven by SND related processes^18^. 48 h after stroke we began the LOPC protocol, which involved exposing male mice to normobaric 11% oxygen for 8 hours/day for 14 days (Fig. 1). Our previous studies have shown that SND become apparent in the thalamus after 14 days from stroke, specifically in the thalamic regions posterior complex (PO) and ventral posterolateral nucleus (VPL) which are connected to the motor-sensory cortical area. Therefore we focused our intervention within this time window and we investigated whether LOPC treatment disturbed NMDAR mediated excitotoxicity. As SND is a dynamic process, we also extended our observations to include an additional time point at 30 days post stroke. The aim of including this latter time point was to verify how SND and associated microgliosis evolved over time and if LOPC effects were maintained.

## Results

### LOPC prevents thalamic neuronal death

**Figure 2:**
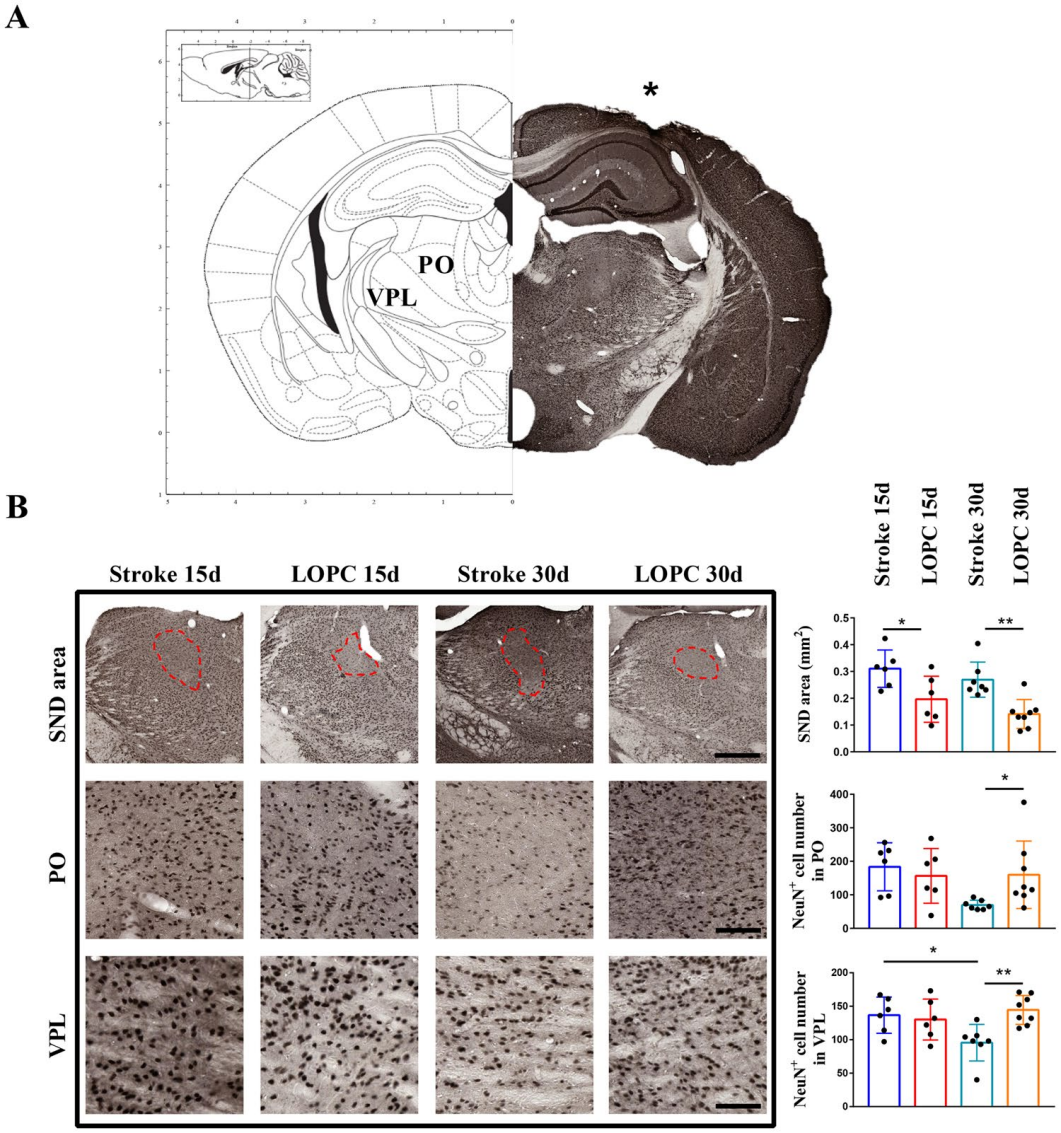
Secondary neuronal death is prevented by LOPC at 30 days. (**A**) Brain map adapted from^47^ and exemplificative NeuN staining showing the infarct region (*) and the thalamic regions VPL and PO. (**B**) Identified area of PO SND (dash line, scalebar = 400 μm) and representative images of the PO (scalebar = 70 μm) and VPL (scalebar = 40 μm) areas used for NeuN^+^ cells count. On the right, quantification shows that LOPC decreases the area of SND in PO and prevents NeuN^+^ cells decrease in the VPL and PO. Results are shown as the mean ± SD. *p < 0.05, **p < 0.01, 2-way ANOVA with Tukey’s multiple comparison test.

In order to investigate the neuroprotective activity of LOPC, we estimated the number of neurons by counting NeuN+ cells in thalamic posterior (PO) and ventral posterolateral nuclei (VPL) (Fig. 2A). We also evaluated neuronal loss in the PO also by measuring the area of NeuN+ cell loss (Fig. 2B). The area of SND in VPL could not be evaluated since the neuronal loss wasn’t as evident as in the PO. Data show that LOPC group at 30 days have significantly more NeuN^+^ cells in thalamus (p < 0.01) and inside the regions VPL (p < 0.01) and PO (p < 0.05), and that the area of PO affected by SND was smaller after LOPC both at 15 and 30 days (p < 0.05 and p < 0.01 respectively).

### Thalamic inflammation is resolved at 1 month after LOPC

**Figure 3:**
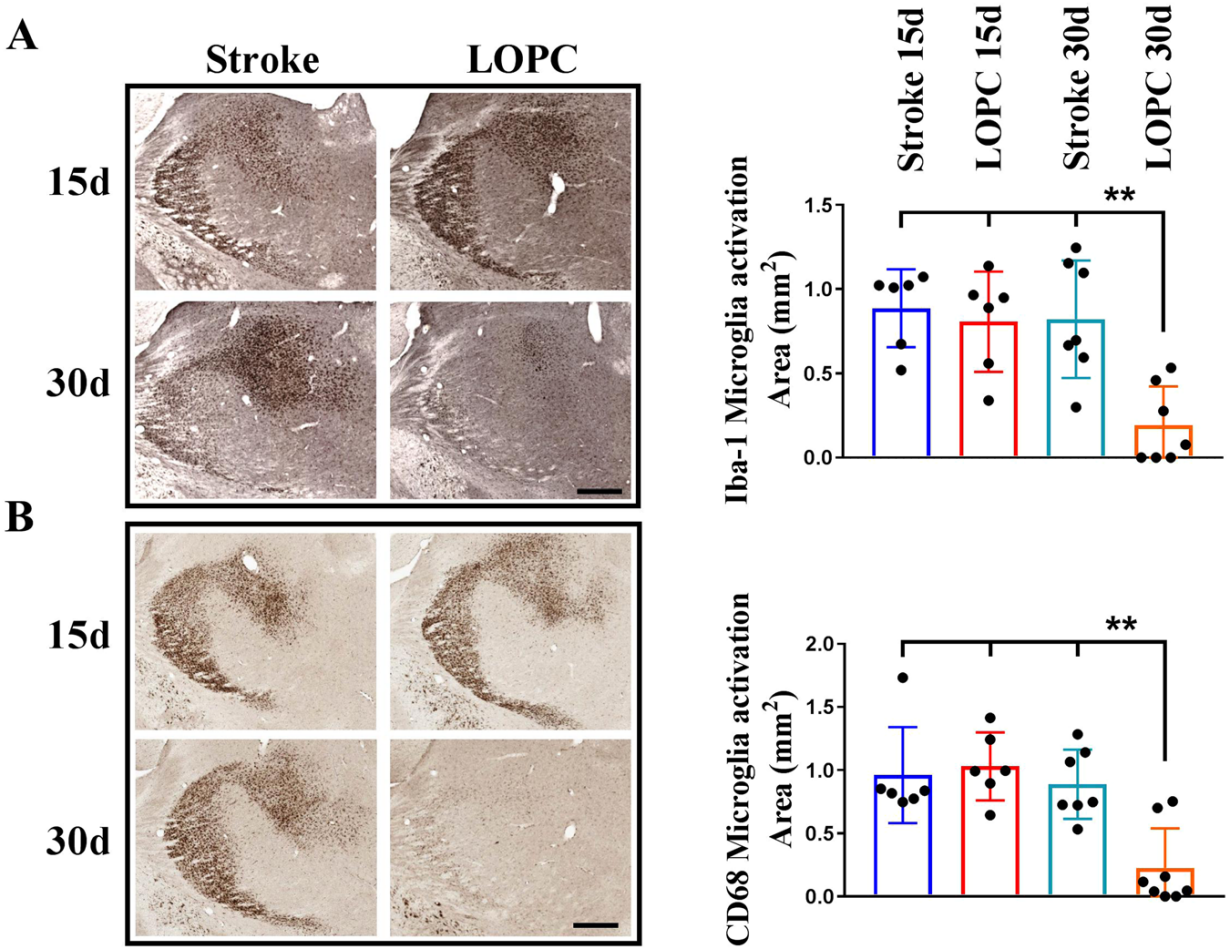
Thalamic microglia activation is resolved at 30 days after LOPC. (**A**) Decreased microglia activation area in thalamus of LOPC mice at 30 days, as estimated by Iba1 and CD68 (**B**) staining (scalebar = 300 μm). **p < 0.01, 2-way ANOVA with Tukey’s multiple comparison test.

**Figure 4:**
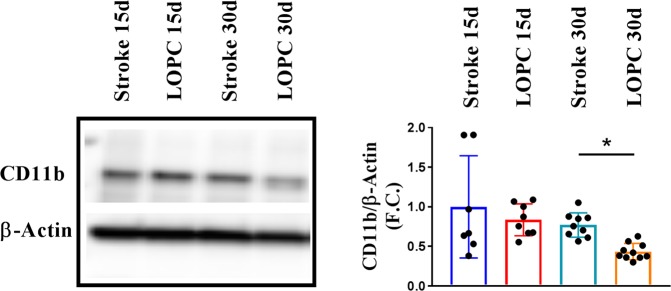
Thalamic microglia expression of inflammatory marker CD11b. Representative western blot showing changes in CD11b expression (n = 8–10). Quantification revealed decreased expression of CD11b in LOPC mice at 30 days. Results are shown as the mean ± SD. *p < 0.05, 2-way ANOVA with Tukey’s multiple comparison test.

Regions of secondary damage are associated with extensive microgliosis. Therefore we evaluated microglia activation in the thalamus by investigating changes in the immunohistochemical expression of Iba1 (Fig. 3A) as well as CD68 (Fig. 3B). We further cross-validated these findings using western blot quantification of another marker of microglial ‘activation’, CD11b (Fig. 4). Analysing the thalamic area we observed extensive microgliosis at 15 days post stroke in both stroke and LOPC groups, while at 30 days microglia activation was significantly decreased in LOPC treated (Iba1 p < 0.01, CD68 p < 0.01, CD11b p < 0.05). 2-way ANOVA analysis of the % of thresholded material showed that Iba1 (at pixel intensity 50) and CD68 (pixel intensity 100) decrease in the VPL area, with significant contribution from both time (Iba1 p < 0.0001, CD68 p < 0.001) and LOPC (p < 0.05 both Iba1 and CD68) (Supp. Figs 1 and 2). Instead in the PO area, only LOPC treatment was found giving a significant contribution (p < 0.05), with a significant difference between stroke and LOPC at 30 days for both Iba1 and CD68 (p < 0.05) as assessed by post-hoc analysis.

### Thalamic calcium accumulation is not affected by LOPC

**Figure 5:**
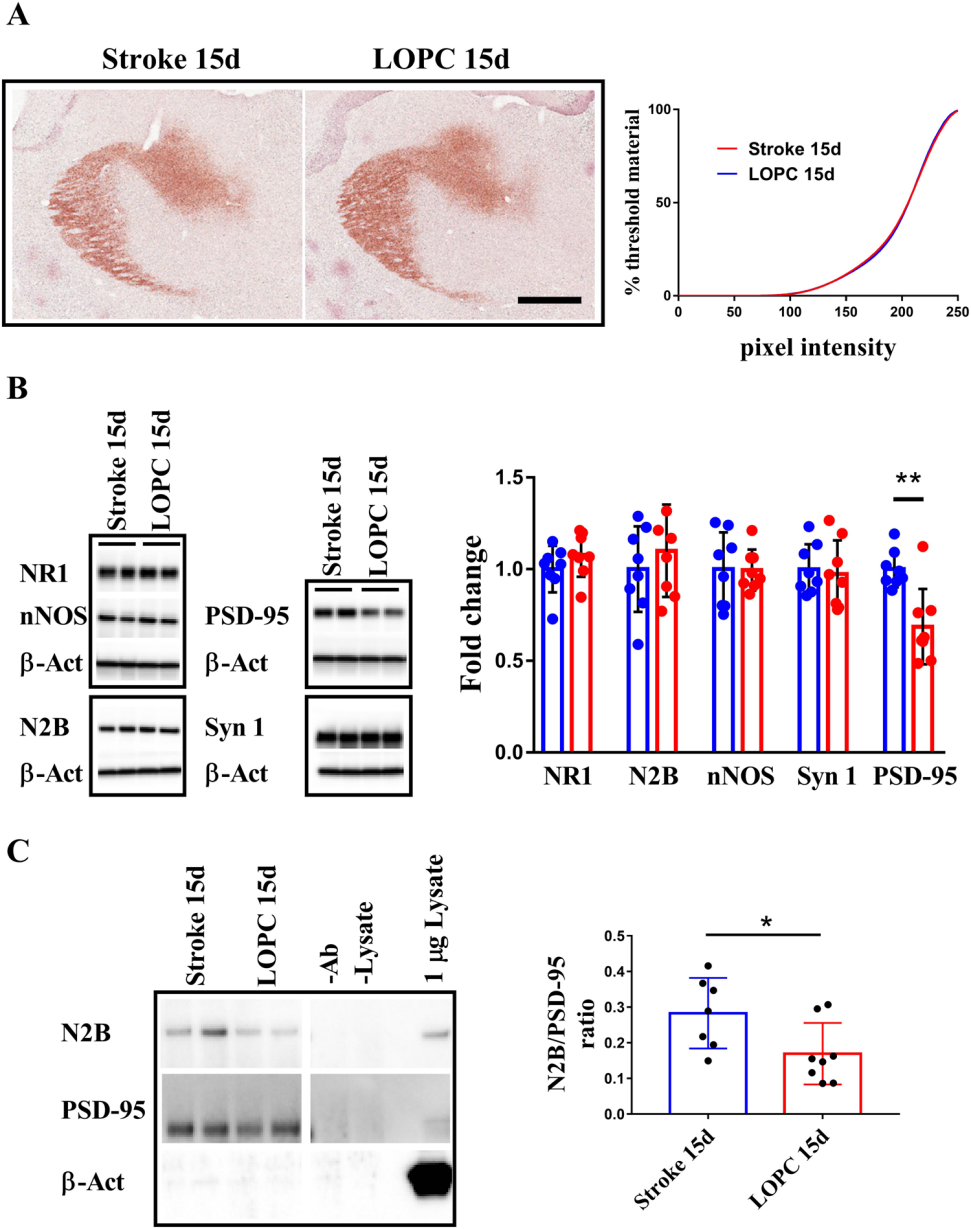
NMDAR mediated nNOS activation is prevented by LOPC at 15 days. (**A)** Calcium accumulation in the thalamus is not affected by LOPC at all pixel intensities as shown by cumulative tresholding analysis (scalebar = 400 μm). (**B**) The thalamic expression NR1, N2B and nNOS do not change in time or with treatment as assessed by WB. At 15 days, WB analysis show that the expression of synaptic marker PSD-95 is decreased by 50% in LOPC while Synapsin 1 expression is unchanged. (**C**) Co-immunoprecipitation of N2B and PSD-95 shows decreased interaction in LOPC samples between NMDAR and PSD-95 at 15 days (-Ab: beads not coated incubated in lysate; -Lysate: beads coated incubated in PBS). Images cropped from Supp. Fig. 3. Results are shown as the mean ± SD. *p < 0.05, Mann Whitney U test.

Calcium induced excitotoxicity has been proposed as potential cause of thalamic SND, therefore we evaluated by Alizarin red S staining the accumulation of calcium in the thalamus after 15 days post stroke^19^. We could not find any difference in the calcium accumulation between stroke and LOPC at 15 days (see, Fig. 5A).

### Decreased NMDAR/PSD-95 interaction after LOPC treatment

We analysed in the thalamus the expression levels of the components of the complex NMDAR/PSD-95/nNOS that can mediate calcium excitotoxicity. In particular NMDAR is composed by one NR1 and 2 NR2 (N2A and/or N2B) subunits, being N2B the subunit interacting with PSD-95. We could not find any statistical evidence to support the notion that LOPC altered the expression of NR1, N2B or nNOS (Fig. 5A). We did, however, identify that LOPC induced a significant decrease in PSD-95 expression (p < 0.01) (Fig. 5B). To verify if there was a disturbance in synapse formation we also quantified the expression of Synapsin1 but we couldn’t find any statistical evidence to support a LOPC mediated change, suggesting that the decrease in PSD-95 was specific. Next we determined whether decreased PSD-95 expression involved decreased PSD-95/NMDAR interaction as determined via co-immunoprecipitation of PSD-95 with N2B subunit. PSD-95 was successfully pulled down, as we verified by using a different PSD-95 antibody to probe the membrane, and co-immunoprecipitated specifically with N2B (Fig. 5C, left panel). We ruled out that the signal could be due to non specific binding to the beads since β-actin was neither detectable in CO-IP samples nor in samples of beads not antibody-coated incubated with same amount of lysates (-Ab lane, Fig. 5C right panel). As further control antibody-coated beads not incubated with lysates did not give any PSD-95, N2B or β-actin signal (-Lysate, Fig. 5C). The results showed a decreased co-immunoprecipitation of PSD-95 with N2B in LOPC samples (p < 0.05). This experiment was replicated with similar results.

### LOPC ameliorates white matter disturbances

Since SND is a disconnection injury we evaluated white matter tract alterations using Sudan Black staining of myelin. We found a significant decrease in white matter structural loss in corpus callosum at Bregma −1.5, as a difference between the contralateral (CL) and ipsilateral (IL) hemispheric area (mm^2^), at 15 days post stroke in LOPC mice (p < 0.05). The LOPC protective effects of white matter disturbances were sustained at 30 days post-stroke (p < 0.05) (Supp. Fig. 4).

## Discussion

The specificity and precision of occlusion that can be induced via use of photothrombosis, makes it an ideal model to study secondary neurodegeneration. Our group and others have used this model extensively to study the progression of secondary neurodegeneration in the thalamus. The thalamus is recognised to be mono-synaptically connected to the motor and somatosensory corticies but is not directly affected by the primary occlusion^20–22^. In the current study we were interested in exploring a specific question, to what extent could low oxygen post-conditioning (LOPC) influence the severity of thalamic degeneration, and in particular whether it could modulate the excitotoxic processes within the degenerating thalamus. In short, we observed a striking ability of LOPC to induce robust neuroprotection within the thalamus in terms of mature neurons cell count. Importantly, we also have observed, for the first time, that LOPC reduced the interaction of PSD-95 with the N2B subunit of NMDAR, a key interaction in eleciting excitotoxic cell death. These results provide novel evidence that one of the potential mechanisms underlying LOPC neuroprotection may be a reduction in exitotoxicity mediated by the NMDAR-PSD-95-nNOS pathway.

To quantify the extent of neuroprotection offered by LOPC we used two different but complementary measurements to estimate the extent of secondary thalamic tissue loss: the area of thalamic injury that is affected by SND and the degree of neuronal loss in that area. Both these methods have previously been used to identify thalamic neuronal death^20,23–25^. When considering the area of loss we observed that the SND area in the thalamus did not significantly change over time between 15 and 30 days. This suggests that in our model the event defining the perimeter of SND has largely developed within 15 days after stroke, an observation that was mirrored in the LOPC group. Our NeuN^+^ cell count results, however, suggested that loss of cells within this perimeter continued to increasing over time between 15 and 30 days (NeuN^+^ cell count, PO 183.5 vs 69.5, VPN 136.6 vs 95.5), an observation that also aligns with^26^. Based on these results and other studies^27^, we conclude that NeuN^+^ cell counts represent a more sensitive indicator of SND than area measurement, and deduce that the SND takes some time to fully manifest in the model of ischemic injury. The progressive nature of the cell death could be driven by a number of mechanisms but is most likely caused by the spreading of death by apoptosis over time^28^ which is consistent with an excitotoxic mechanism^29,30^. Of note, in the LOPC group we did not observe further neuronal death after 15 days. This result suggests that 2 weeks of LOPC was sufficient to constrain those processes that contribute to neuronal loss and appear to significantly slow its progression. Further, the results from Sudan Black staining suggest that LOPC provides protective effects on white matter structural loss.

Microgliosis, in conjunction with selective neuronal loss, is recognised to be another characteristic feature of SND^31^. Our results show that SND was associated with a considerable change in microglial morphology (Fig. 3A) as well as enhanced expression of CD68, a putative marker of phagocytosis (Fig. 3B) and CD11b a putative marker of complement recognition (Fig. 4) at day 15 post stroke. These changes are highly consistent with a phenotype observed in microglia that are responding to injury and damage within the CNS^32^ and were confirmed by analysis of the immunolabelling intensity (Supp. Figs 1 and 2). LOPC was observed to significantly reduce microglial ‘activation’ on day 30 (15 days after the end of LOPC treatment) but not earlier. We find this observation particularly interesting when taken in conjunction with our neuronal loss findings. As the benefit of LOPC on neuronal loss was already evident by day 15 and as there was no measurable LOPC effect on microglia until 30 days, it would suggest that the benefits of LOPC are not primarily attributable to modulation of microglial activity.

Since we observed a direct effect on LOPC at 15 days on thalamic cell loss we next evaluated the involvement of LOPC on excitotoxicity at this time point. We first confirmed an accumulation of calcium within the ipsilateral thalamus (Fig. 5A), effectively establishing the possibility that calcium-dependent excitotoxicity was occurring within this region. We could not identify any differences between stroke alone and LOPC treated animals in calcium build up. We next considered the expression levels within the thalamus of the main elements involved in the excitotoxic axis: NMDAR, PSD-95 and nNOS. Here we identified a significant and specific decrease of 30% in PSD-95 expression after LOPC compared to stroke alone at 15 days (Fig. 5B). We considered the reduction in PSD-95 to be specific and not likely due to altered plasticity, since the level of Synapsin-1 was not altered. This decrease suggested a potential alteration of the excitotoxic signalling initiated by NMDAR, as the crucial event for this signalling cascade is the binding of PSD-95 to NMDAR. Therefore, we next investigated by co-immunoprecipitation (CO-IP) the levels of interaction between the PSD-95 and the NMDAR subunit N2B^33^. The results showed that in tissue obtained from the ipsilateral thalamus of mice exposed to LOPC there was a lower level N2B being pulled down with PSD-95 (50% decrease, Fig. 5C). As we could find no evidence of non-specific proteins such as β-actin on the pull down, we are inclined to conclude that LOPC reduces also NMDAR and PSD-95 interaction, a finding that is consistent with the ability of LOPC to dampen the severity of excitotoxicity.

While we have found that the neuroprotective effect of LOPC may be mediated via an effect of excitotoxicity it is important to recognise that LOPC may effect neuroprotection via numerous mechanisms. For instance it has been shown that LOPC promotes post-translational modification of neuroprotective heat shock protein^34^, inactivation of the ERK pathway^35^ and promotion of VEGF signalling^36^. Although previous work has already suggested that exposure to low oxygen environments may dampen NMDAR mediated activation of nNOS^37^, to our knowledge this is the first report proposing to exploit LOPC to interfere with the excitotoxic signalling leading to SND, by decreasing PSD-95 expression^10^ and the interaction between NMDAR and PSD-95. While LOPC has been shown to be safe in humans^11^, more studies are needed to identify potential unwanted effects of this treatment after stroke.

In conclusion we have shown that LOPC can prevent stroke-associated secondary neuronal loss within the thalamus by interfering with the excitotoxic process mediated by the NMDAR/PSD-95 interaction. However, further studies are required to dissect the mechanism by which the excitotoxic signal originating from the primary injury can induce SND and define its contribution to distal neuronal death. In this study, we focused on the sub-acute phase, commencing low oxygen exposure 48 hours after stroke, when the primary damage is mostly developed. Considering that in the mouse the peak of SND occurs at one month after ischemia, it was vital to begin the treatment within this time window. The time scale for humans is quite different, since distal neuronal death and microglia activation lasts for months after stroke^38,39^. In a translational perspective, the efficacy of LOPC at later time still needs to be validated. In addition to duration of effect further consideration needs to be given to issues including the optimal oxygen concentration^40^, atmospheric pressure^41^, dosage^42^ as well as consider the interaction of the treatment with common comorbidities^43,44^. At this stage, however, LOPC is a promising treatment with theoretically few side effects and an excellent candidate for translational approaches.

## Materials and Methods

### Procedures

Animals were obtained from the Animal Services Unit at the University of Newcastle. Experiments were approved by the University of Newcastle Animal Care and Ethics Committee and conducted in accordance with the New South Wales Animals Research Act and the Australian Code of Practice for the use of animals for scientific purposes.

### Stroke

On day 0 mice were anesthetised by using 2% of isoflurane followed by intraperitoneal injection of 0.2 ml rose Bengal at 10 mg/ml concentration. After eight minutes a cold light source with a fibre optic end of 4.5 mm diameter was placed at 2.2 mm left lateral to Bregma onto the exposed skull for 15 minutes (Fig. 1)^20^. Mice were excluded if negative for stroke by histological evaluation at the relative endpoint. The sample size was estimated based on previous experiments (data not shown).

### LOPC

48 hours after stroke induction (day 0), a total of 80 mice were randomly allocated to control (atmospheric oxygen) or LOPC group. LOPC was delivered using a customized hypoxic environment IVC rack provided 11% oxygen for 8 h/day, at atmospheric CO_2_ concentration (300 ± 50 ppm) and atmospheric pressure (100 kPa).Following 14 days half of each group was euthanized for tissue collection (n. 6–10 depending on experiment). The remaining half of both groups was continuously exposed to atmospheric air for further 14 days before the endpoint (day 30).

### Tissue processing

At the scheduled endpoint (day 15 or 30) mice were deeply anaesthetised with sodium pentabarbitol and perfused via the ascending aorta with ice cold PBS followed by ice cold 4% paraformaldehyde (pH 7.4) for immunohistochemical analysis or with cold PBS only for western blotting. For immune-histochemical analysis, brains were dissected, post-fixed in 4% paraformaldehyde for 4 h and transferred to 12.5% sucrose in PBS for storage for a maximum of 1 month. Coronal sections of the brains were sectioned with a freezing microtome (Leica) at a thickness of 30 µm. For cohorts dedicated to western blot analysis, brains were dissected and flash-frozen in −80 °C isopentane. Frozen brains were sliced using the cryostat at a thickness of 200 µm. Tissue was then punched using 2 mm tissue punch in the thalamus region (Bregma −1.2 to −2.2). Samples were stored frozen in −80 °C until further analysis.

### Western blot

Thalamic samples were sonicated in 150 µl lysis buffer (50 mM TRIS buffer pH 7.4, 1 mM EDTA, 1 mM DTT, 80 µM ammonium molybdate, 1 mM sodium pyrophosphate, 1 mM sodium vanadate, 5 mM b-glycerolphosphate, 1 protease inhibitor cocktail tablet, 1 phosphatase inhibitor cocktail tablet, final concentration) and centrifuged for 20 min at 4 °C. Next, supernatants were collected and protein levels were estimated by Pierce BCA protein assay kit according to the manufacturer’s instructions. 15 µg of lysate were loaded per lane. After transfer and blocking, the membranes were probed with the appropriate antibody: CD11b (Cat#ab75476, Abcam), N2B (Cat#14544, Cell Signalling), NeuN (Cat#MAB377, Millipore), nNOS (Cat#4236, Cell Signalling), NR1 (Cat#5704, Cell Signalling), PSD-95 (Cat#3409, Cell Signalling), Synapsin 1 (Cat#5297, Cell Signalling), β-actin (Cat#A3854, Sigma-Aldrich). Analysis was performed with the Amersham Imager 600 Analysis Software.

### Immunohistochemistry

Free-floating sections were immunostained as described with minor modification. All reactions for labels marker were run at the same time, with same reagents, at the same concentrations. Briefly, brain sections were incubated with 1% hydrogen peroxidase for 30 min at 25 °C and followed by 3% horse serum for 30 min at 25 °C. Incubation with primary antibodies was performed for 72 h at 4 °C followed by incubation with the proper secondary antibody for 1 h at 25 °C. Next, brain sections were incubated for 2 h at 25 °C with avidin–biotin-peroxidase complex and finally developed using DAB peroxidase substrate. Brain sections were washed with PBS in between each incubation step. After processing was complete, sections were mounted onto polylysine coated slides and cover slipped. For calcium staining, brain sections were mounted on polylysine coated slides and incubated in 2% Azalin Red S (Sigma) in distilled water (pH 4.1) for 30 seconds^19^, dehydrated and cover slipped. Sudan Black B (Sigma-Aldrich, USA) protocol was performed as previously described^45^. Briefly, sections were mounted and rinsed with 70% ethanol followed by 15 min incubation with Sudan Black B solution. After staining sections were rinsed with 70% ethanol and water and 5 min counterstained with nuclear fast red solution (Sigma-Aldrich, USA). Images were taken at 20X with an Aperio AT2 (Leica). ImageJ software (1.50, NIH) or Matlab custom script (R2015a, MathWorks) were used to measure NeuN^+^ cell number and area coverage of immunolabelling by cumulative tresholding^46^. The estimated corpus callosum loss area was determined using ImageJ software [area of contralateral hemisphere - area of ipsilateral hemisphere].

### Co-IP

n = 16 mice underwent stroke procedure and after 48 hours were randomly allocated to atmospheric oxygen or LOPC for 14 days. One mouse was excluded since negative for stroke by histological evaluation. The tissue was collected as for the western blot procedure. The thalamic area was punched and tissue was gently homogenised with a pestle in 50 mM tris, 150 mM NaCl and 1% Triton-X 100 with protease inhibitor cocktail (Roche). Protein concentration was quantified by Pierce BSA assay. Beads from Dynabeads™ Protein A Immunoprecipitation Kit (Invitrogen) were washed 3x in PBS-T 0.05% and incubated with PSD-95 antibody (Cat#2507, Cell Signalling) at 1:50 concentration in a volume of 800 ul for 3 hours in rotation at RT. After 2 washes in PBS-T, beads were resuspended in Lysis buffer and an equivalent amount of 50 ul of starting beads was added to 75 ug of proteins in a final volume of 400 ul and incubated by rotation O.N. at 4 °C. The following day beads were washed 2 times in Lysis buffer changing the tube, spinned down, resuspended in 15 ul of Laemmli buffer and boiled for 10 minutes. Finally the samples were centrifuged 4000 g for 3 minutes to pellet the beads and loaded. Blot was hybridised with antibodies for PSD-95 (Cat#3409, Cell Signalling), N2B (Cat #14544 Cell Signalling) and β-Actin. Beads coated with the N2B antibody successfully pulled down the target protein but failed to coimmunoprecipitate PSD-95 (not shown).

### Statistics

Data were analysed with either Mann-Whitney U test or 2-way ANOVA followed by Tukey’s multiple comparison post-test using Prism 7 (GraphPad). P < 0.05 was considered significant.

## Supplementary information

Supplementary figures
