# Supplementary material for: Low oxygen post conditioning prevents thalamic secondary neuronal loss caused by excitotoxicity after cortical stroke
Source: Sci Rep. 2019 Mar 19;9:4841. doi: 10.1038/s41598-019-39493-8 (PMC6425023; doi:10.1038/s41598-019-39493-8)
Supplement: Supplementary file 1 — Supplementary figures [file 41598_2019_39493_MOESM1_ESM.pdf]

# Low Oxygen Post Conditioning prevents thalamic secondary neuronal loss caused by excitotoxicity after cortical stroke

***Authors:***

Giovanni Pietrogrande, Katarzyna Zalewska, Zidan Zhao, Mahmoud Abdolhoseini, Wei Zhen Chow, Sonia Sanchez-Bezanilla , Lin Kooi Ong, Sarah J Johnson, Michael Nilsson, Frederick R Walker

## *Supplementary Information*

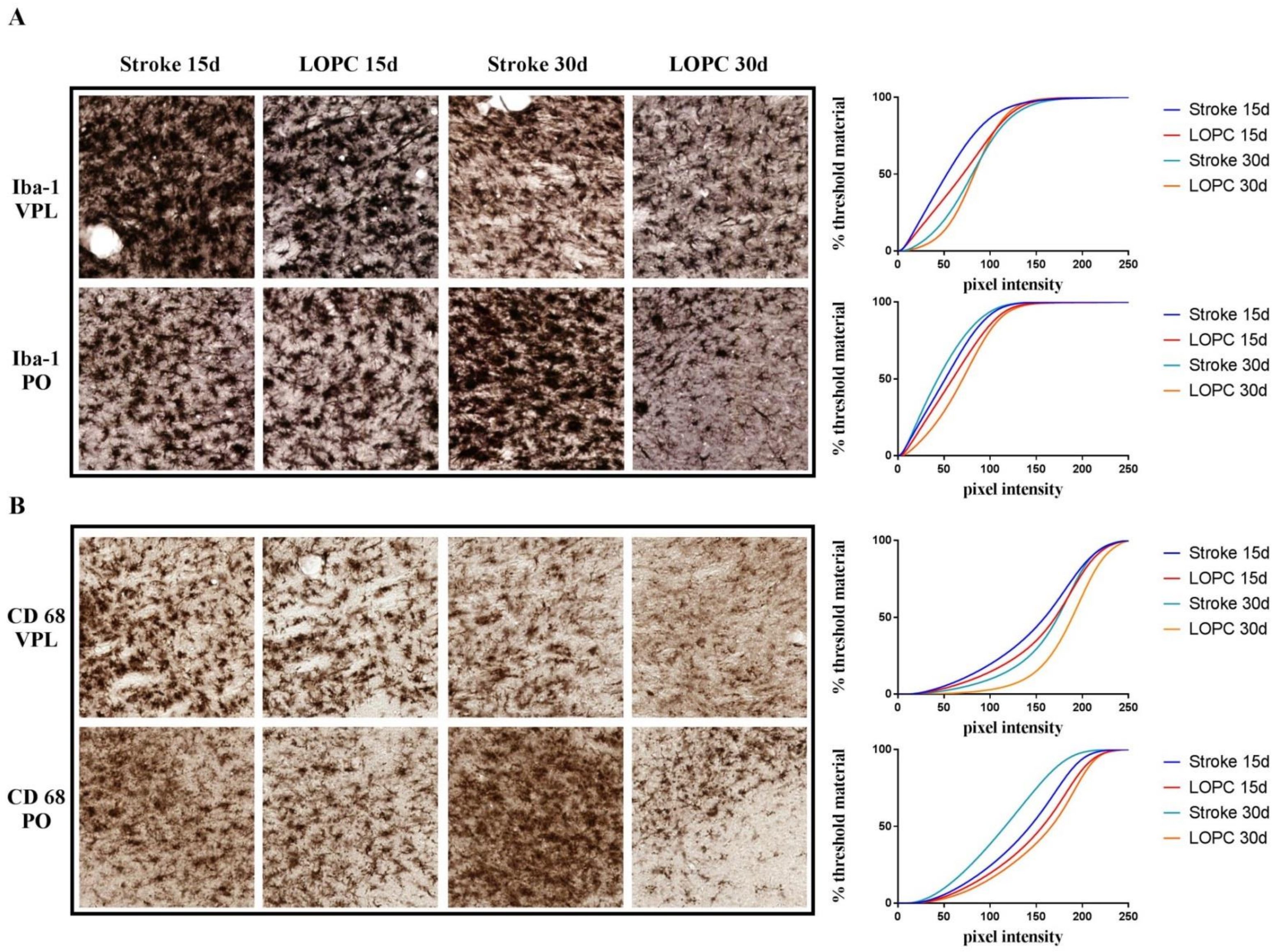
A
Stroke 15d
LOPC 15d
Stroke 30d
LOPC 30d
Iba-1 VPL
Iba-1 PO
B
CD 68 VPL
CD 68 PO
% threshold material
pixel intensity
0
50
100
150
200
250
Stroke 15d
LOPC 15d
Stroke 30d
LOPC 30d

**Supp Fig 1. Quantification of immunolabelling for Iba1 and CD68 in the PO and VPL.** Cumulative thresholding analysis of the intensity of immunolabelling confirms general decrease in the levels of microglia activation at 30 days after LOPC compared to stroke alone.

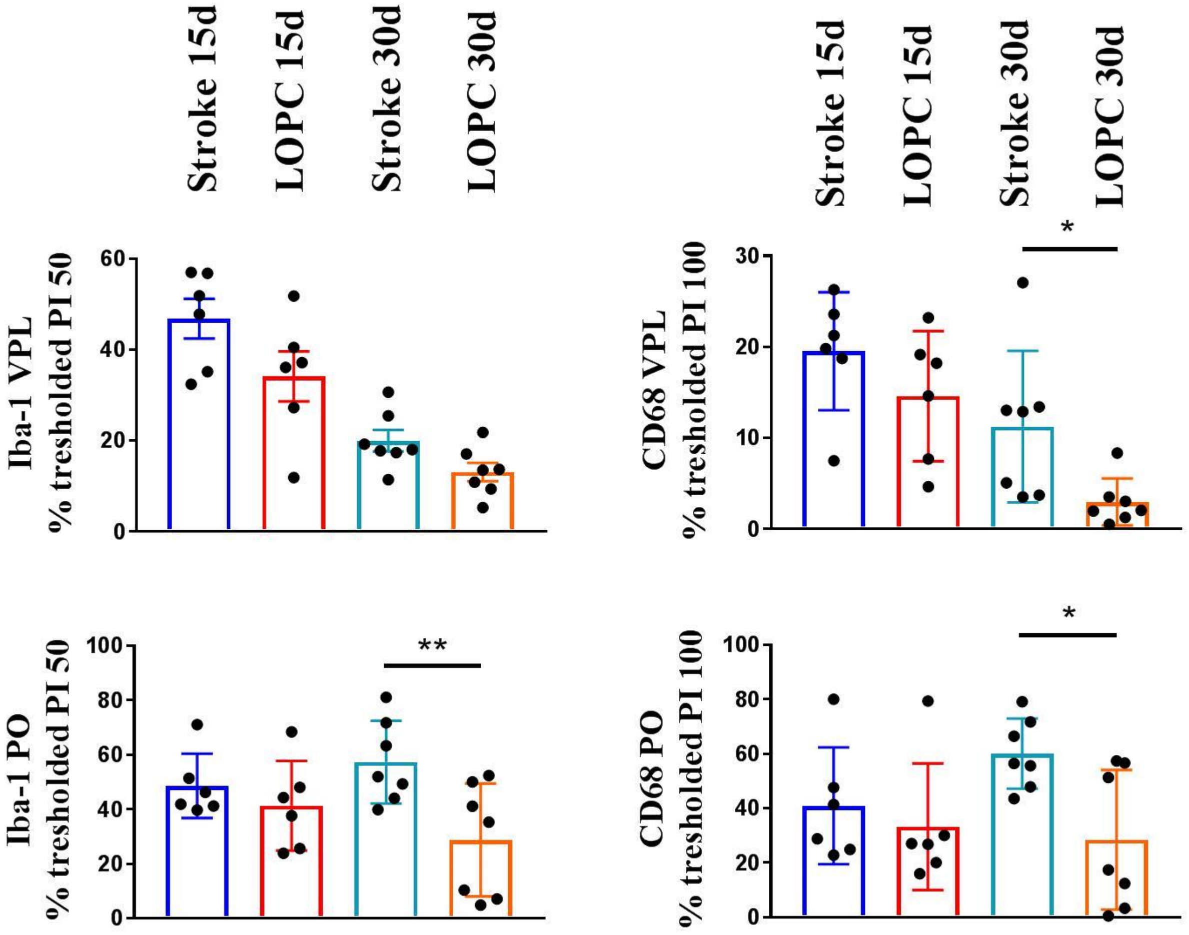


**Supp Fig 2. Specific quantification of cumulative thresholding.** Analysis of the thresholded material of Iba-1 and CD68 staining in the VPL and PO. The optimal pixel intensity (PI) was assessed manually.

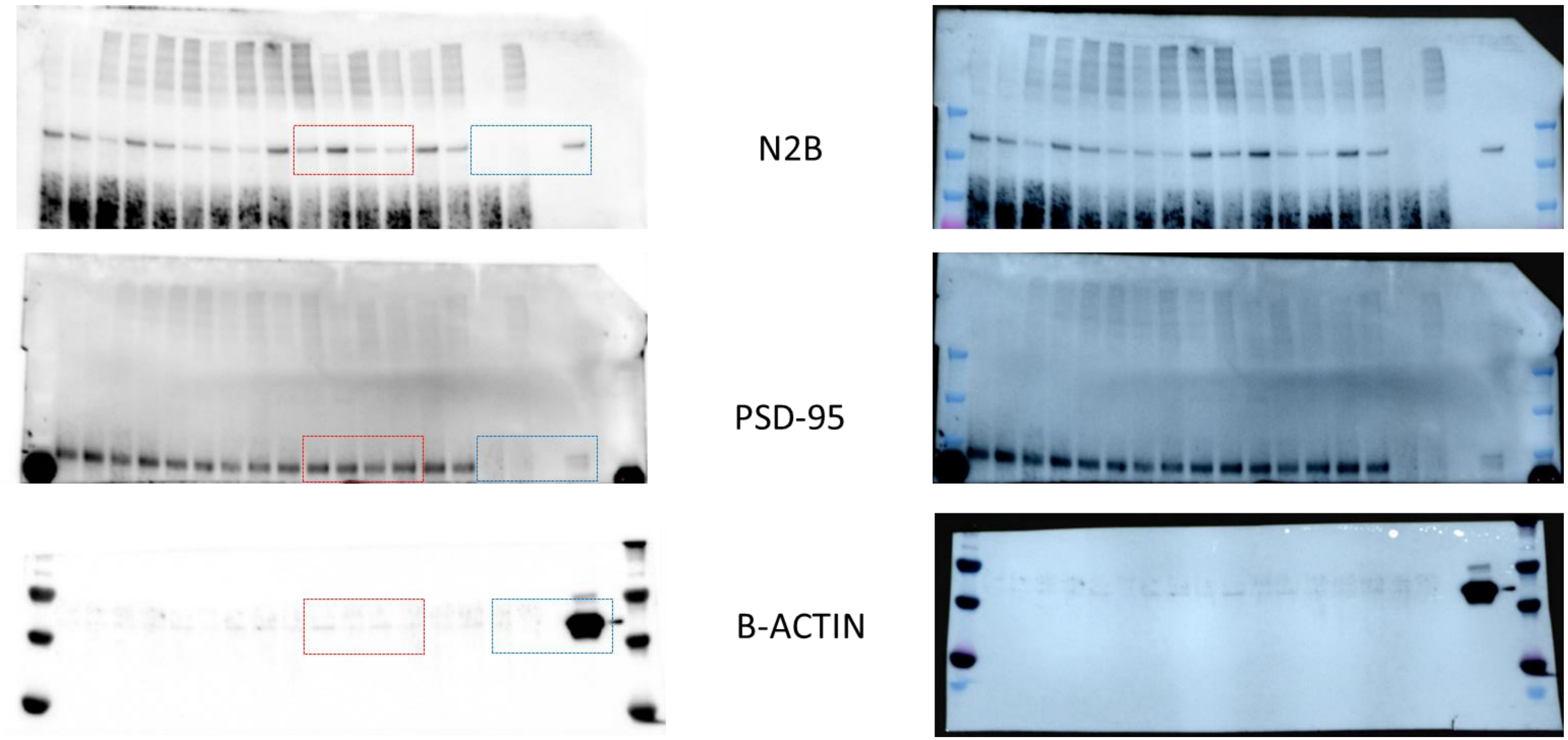
N2B
PSD-95
B-ACTIN

**Supp Fig 3. CO-IP original western blot.** Picture of the original western blot cropped for Fig. 5c and overlay with white light for molecular weight marker (Bio Rad, Cat# 161-0374) visualization. Loading order: Stroke1, Stroke2, LOPC1, LOPC2, Stroke3, Stroke4, LOPC 3, LOPC4, Stroke5, Stroke6, Stroke7, LOPC5, LOPC6, LOPC7, LOPC8, -Ab, -Lys, empty lane, 1µg Stroke thalamus lysate. Red box highlights the left part of panel Fig 5c, blue box the right panel of Fig 5c.

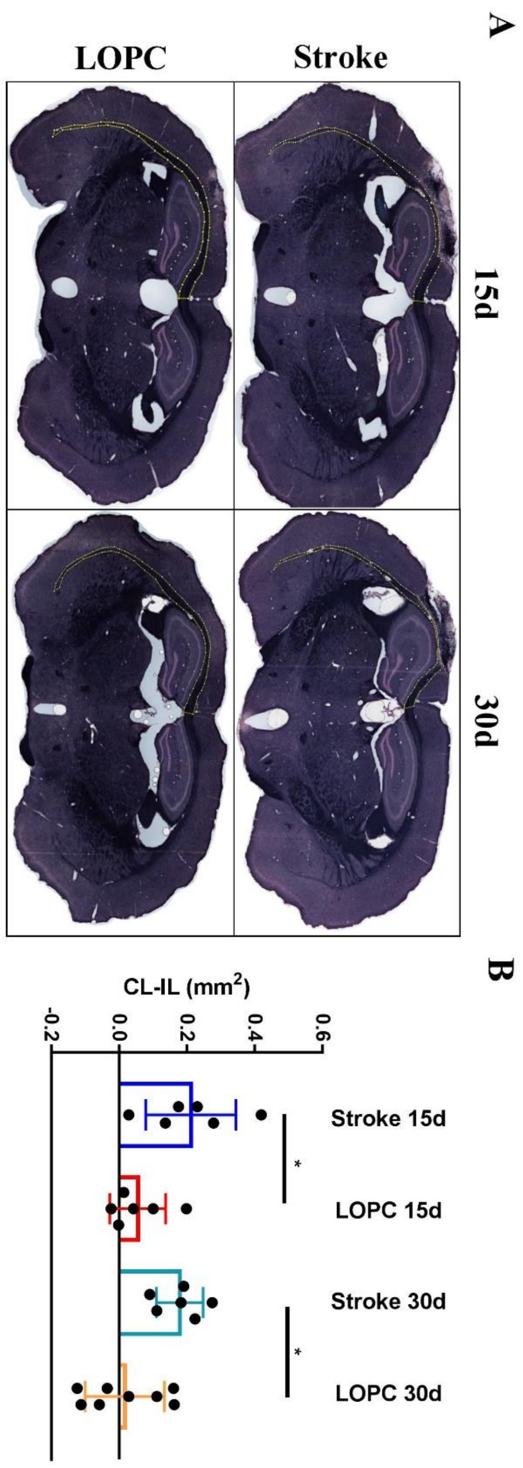


**Supp Fig.4 LOPC ameliorates white matter loss. (A)** Sudan Black B staining at Bregma levels -1.5. Yellow line represents the corpus callosum traced using ImageJ. **(B)** Quantification of corpus callosum area measured as a difference between the contralateral (CL) and ipisilateral (IL) hemisphere revealed a significant decrease in white matter structural loss at both 15 and 30 days post-stroke. Results are shown as the mean ± SD. * $p<0.05$, Mann Whitney U test.
